# Aggressive breast cancer in western Kenya has early onset, high proliferation, and immune cell infiltration

**DOI:** 10.1186/s12885-016-2204-6

**Published:** 2016-03-10

**Authors:** Rispah T. Sawe, Maggie Kerper, Sunil Badve, Jun Li, Mayra Sandoval-Cooper, Jingmeng Xie, Zonggao Shi, Kirtika Patel, David Chumba, Ayub Ofulla, Jenifer Prosperi, Katherine Taylor, M. Sharon Stack, Simeon Mining, Laurie E. Littlepage

**Affiliations:** University of Notre Dame, Notre Dame, IN USA; Harper Cancer Research Institute, University of Notre Dame, 1234 N Notre Dame Avenue, South Bend, IN USA; Moi University, Eldoret, Kenya; Maseno University, Maseno, Kenya; Indiana University School of Medicine, Indianapolis, IN USA; Eck Institute for Global Health, Notre Dame, IN USA; Indiana University School of Medicine-South Bend, South Bend, IN USA

**Keywords:** Kenya, Breast cancer, Estrogen receptor, CD163, CD25

## Abstract

**Background:**

Breast cancer incidence and mortality vary significantly among different nations and racial groups. African nations have the highest breast cancer mortality rates in the world, even though the incidence rates are below those of many nations. Differences in disease progression suggest that aggressive breast tumors may harbor a unique molecular signature to promote disease progression. However, few studies have investigated the pathology and clinical markers expressed in breast tissue from regional African patient populations.

**Methods:**

We collected 68 malignant and 89 non-cancerous samples from Kenyan breast tissue. To characterize the tumors from these patients, we constructed tissue microarrays (TMAs) from these tissues. Sections from these TMAs were stained and analyzed using immunohistochemistry to detect clinical breast cancer markers, including estrogen receptor (ER), progesterone receptor (PR), human epidermal growth factor 2 receptor (HER2) status, Ki67, and immune cell markers.

**Results:**

Thirty-three percent of the tumors were triple negative (ER-, PR-, HER2-), 59 % were ER+, and almost all tumors analyzed were HER2-. Seven percent of the breast cancer patients were male, and 30 % were <40 years old at diagnosis. Cancer tissue had increased immune cell infiltration with recruitment of CD163+ (M2 macrophage), CD25+ (regulatory T lymphocyte), and CD4+ (T helper) cells compared to non-cancer tissue.

**Conclusions:**

We identified clinical biomarkers that may assist in identifying therapy strategies for breast cancer patients in western Kenya. Estrogen receptor status in particular should lead initial treatment strategies in these breast cancer patients. Increased CD25 expression suggests a need for additional treatment strategies designed to overcome immune suppression by CD25+ cells in order to promote the antitumor activity of CD8+ cytotoxic T cells.

**Electronic supplementary material:**

The online version of this article (doi:10.1186/s12885-016-2204-6) contains supplementary material, which is available to authorized users.

## Background

Breast cancer is the most frequently diagnosed and the most deadly cancer among women worldwide, taking roughly half a million lives per year [1]. Between 1980 and 2010, the global rate of breast cancer incidence increased 2.6 times (i.e., from 641,000 to 1,643,000 patients) [2]. Unfortunately, the global rates of breast cancer incidence and mortality continue to increase, particularly in developing countries [2]. In fact, 59 % of the worldwide breast cancer deaths is estimated to occur in developing countries [1].

Similar to global cancer trends, breast cancer is the most highly diagnosed and leading cause of cancer deaths in women throughout Africa (63,100 deaths in 2012) [3]. However, in Africa, noncommunicable diseases like cancer are not considered as pressing of a burden to society as infectious diseases, which have a higher prevalence in the patient population. Limited resources for surveillance, treatment, and research, as well as low public awareness campaigns for early detection and treatment affect the rate of cancer diagnosis. In addition, most standard care and treatments used globally for treating breast cancer are derived from research on patient populations in resource-rich developed countries, which results in challenging implementation strategies in resource-poor countries [4, 5].

Even within developed countries, breast cancer disease etiology and progression can be quite heterogeneous across patient populations. In contrast to global increases in breast cancer incidence and mortality, the U.S. breast cancer mortality declined as much as 34 % since 1990 [6, 7]. This decline is not consistent across patient groups and varies significantly by race/ethnicity. Non-Hispanic white women have the highest incidence of breast cancer, while African American women have both the highest mortality rate (30.8 deaths per 100,000 females) compared to non-Hispanic white females (22.7 deaths per 100,000) as well as the lowest 5-year cause-specific survival (78.9 %) compared to non-Hispanic whites (88.6 %).

Contributing to the differences in mortality rates, African American women in the U.S. develop breast cancer with a higher grade and a higher representation of early-onset, high-grade, node-positive, and hormone receptor-negative tumors than do patients of other races [8]. A decreased five-year survival rate for African Americans is associated with the pathological presentation at diagnosis but not with patient age or treatment differences [9]. Across age groups, African American women commonly develop tumors that are diagnosed beyond stage I, and African American women who present with stage I disease also have a higher death rate than matched white women [10].

While the striking racial differences in mortality are due in part to differential access to health care for both early detection and treatment of disease, these statistics also reflect the differential incidence of molecular subtypes of breast cancer with poor prognosis across patient populations. Underlying genetic differences across patient populations may harbor unique molecular signatures that result in racial disparities in prognosis and response to treatment. For example, estrogen receptor (ER) status is differentially expressed across racially diverse patient populations. In the U.S., even though African Americans and Hispanics have a higher incidence of ER- breast cancer than is seen in non-Hispanic whites, the majority of African American breast tumors are ER+ [11]. In contrast, tumors from Africa have more heterogeneity. Tumors from breast cancer patients in Nigeria and Senegal were predominantly ER- [12–14], while tumors from another group of patients in Nigeria were predominantly ER+ [15]. Identification of additional molecular markers of breast cancer will help us to understand regional differences that are relevant to disease etiology and treatment.

Of African countries, Kenya has among the highest risk of breast cancer [3]. Breast cancer incidence and mortality rates also have increased significantly since 1980 rates [3]. The goal of our research is to begin to identify underlying molecular mechanisms that promote Kenyan breast cancer by comparing our patient population to patients from Kenya, other parts of East Africa, and African Americans. In this study, we identify the expression patterns of clinical markers in a western Kenyan patient population not studied previously. For this analysis, we selected a patient population from the Moi Teaching and Referral Hospital patient population because previous Kenyan studies looking at the clinical markers of breast cancer were completed in Nairobi, which is a larger metropolis and has different ethnic group demographics, environmental variables, and ethnic groups than the regional areas of Eldoret. A better characterization of the regional differences in breast cancer will guide the creation of early detection programs and effective treatment strategies designed to reduce the cancer mortality rates and suffering in both African and related patient populations.

## Methods

### Patient samples and geographic region

These studies follow appropriate ethical standards and are in accordance with and have been approved by IRBs from both the University of Notre Dame (IRB Approval # 13-06-1102) and Moi University (IRB Approval # 000655). The tissue samples were collected with patient consent at Moi Teaching and Referral Hospital (MTRH), which is the primary academic hospital that serves the entire western Kenya community and is located in the city of Eldoret town (population: 289,380), Uasin Gishu district, North of Rift Valley province of Kenya. Uasin Gishu County is home to 894,179 people. Therefore, the the catchment area for MTRH is a fairly large community and represents a large population. Eldoret is surrounded by agricultural regions and is 330 km northwest of Nairobi. The significant distance between Eldoret and Nairobi makes MTRH a highly utilized hospital facility. As a regional hospital, MTRH treats patients not only from western Kenya but also from eastern Uganda and southern Sudan. Accruing patient samples from one hospital is common in related population-focused studies (e.g., [16–19]).

### Study design

This was a prospective study in which samples were collected consecutively. All breast cancer patients who consented to participation in this study and who attended the MTRH oncology clinic between May 2011 and July 2013 were included in this study (Table 1). The patients included in this study were included in this study after informed consent and had either nonmalignant lumps or clinically established breast cancer (both women and men). The patients had no history of other cancers and no history of chemotherapy prior to diagnosis. The patients who were excluded from the study included patients who did not provide consent, those with a history of other cancer, and those who had a history of treatment with chemotherapy. Using non-cancer tissue from the control population was an important control for the immune cell analysis to provide a baseline comparison of our analysis of the immune cells in cancer tissue to normal tissue in Kenyan breast tissue. In Kenya, breast reduction surgery for cosmetic reasons is uncommon, making it difficult to get true normal tissue. Benign tissues with a normal pathology are typical controls in immune cell quantification analysis when establishing differences in cell populations between cancer vs. not cancer samples [20, 21].

**Table 1:** Clinical characteristics of Western Kenya patient population

	Cancer		Not Cancer	
	N	%	N	%
Cancer status				
Cancer Tissue	68	100	0	0
Not Cancer Breast Tissue	0	0	89	87
Other Tissue	0	0	13	13
Total (N)	68		102	
Gender				
Male	4	7	6	8
Female	54	93	70	92
Total (N)	58		76	
Age at diagnosis				
<40 years	16	30	42	74
40-49 years	11	21	10	18
≥50 years	26	49	5	9
Total (N)	53		57	
HER2 status				
Positive	7	14		
Negative	42	86		
Total (N)	49			
Estrogen receptor status				
Positive	29	59		
Negative	20	41		
Total (N)	49			
Progesterone receptor status				
Positive	19	40		
Negative	29	60		
Total (N)	48			
Triple negative (ER-, PR-, HER2-)	16	33		
Total (N)	48			
Status				
Died	11	69		
Alive	5	31		
Total (N)	16			
Tribe				
Luyha	11	38		
Kalenjin	10	34		
Kikuyu	4	14		
Luo	3	10		
Teso	1	3		
Total (N)	29			
Hormone-based Contraception				
Single agent (Injected or Pill)	11	50		
Combined (Injected and Pill)	2	9		
Neither	9	41		
Total (N)	22			
Marriage				
Married or widowed	24	92		
Not Married	2	8		
Total (N)	26			
Median age (years)	48.5 years (*N* = 38)		31 years old (*N* = 9)	
Mean age (years)	51.9 years (*N* = 38)		35.6 years (*N* = 9)	

Each patient also volunteered clinical data, including a family history of breast cancer or related cancers, saliva was collected, and tumors were obtained from surgical procedures, including mastectomies. Secondary data including HIV status and other medical conditions were extracted from the patients’ files. Incomplete clinical data for the patients was assembled for the patients included in this study. Clinical data was collected from both a questionnaire and from patient records. A questionnaire was administered to all patients who consented to be a part of this study. This enabled collection of the following information: demographic characterization, name, age, gender, nationality, ethnic group, place of birth, village location, county, marital status, weight, and height. Patients also provided information on disease status, when diagnosis was made, treatment, tumor characteristics, left or right, axillary lymph nodes palpability, family history, and risk factors that included age at first menarche, number of pregnancies, breast feeding, use of oral or injective contraceptives, use of HRT, smoking, alcohol consumption, and other environmental factors.

MTRH is the only hospital in western Kenya that is in the AMPATH consoritum. AMPATH promotes care, training, and research as part of its mission and allows “Kenyan leaders to draw upon the resources and talents of North American academic health institutions to tackle the challenges of disease and poverty” (AMPATH website). By being part of the AMPATH consortium, these Kenyan institutions have received extensive training and equipment from these universities. The AMPATH consortium is led by Indiana University and includes multiple universities and academic medical centers in North America.

### Tissue fixation and processing

Harvested specimens were fixed in 10 % neutral buffered formalin, then routinely processed in a Leica TP 1020 tissue processor (Leica Microsystems Inc., Nussloch Gmbh Heidelbeger Nussloch Germany), and paraffin embedded in Paraplast X-tra (McCormick™ Scientific). The embedded tissue blocks were transferred from the MTRH hospital to the University of Notre Dame and submitted for further studies following IRB approval from both institutions. The Kenyan tissue samples were subsequently melted down and re-embedded in Surgipath EM_400 paraffin (Leica Biosystems Inc.), using a Sakura Tissue TEK5 embedding station. Paraffin sections for all studies were cut at 3–4 μm in thickness on a Leica RM2125-RTS rotary microtome for hematoxylin and eosin (H&E) and immunohistochemical staining.

### Pathology

The 3 μm cut tissue slides were stained with Hematoxylin & Eosin (Richard Allan Scientific; Kalamazoo, MI) and submitted for blinded microscopic examinations by a U.S. board certified breast pathologist (S.B.), a Kenyan pathologist (D.C.), and a Ph.D. research pathologist (Z.S.).

### Tissue microarrays

Tissue microarrays (TMAs) were constructed from the cancer and non-cancer breast tissue samples. Tissue cores were punched from donor blocks with a 1 mm diameter stylus and loaded to recipient blocks. Distance between tissue cores was also set at 1 mm. The TMA layout on the recipient TMA blocks was predesigned to represent and distribute randomly across the TMA blocks, the patient heterogeneity (i.e., cancer and non-cancer) as identified by pathology. The specific regions of the blocked tissues selected for the TMA cores were based on the pathology diagnoses from the H&E stained slides. The regions of interest for each block was marked by a pathologist as guidance for core extraction. TMA blocks were constructed with Veridiam Advanced Tissue Arrayer VTA-110CC. Each of the two TMA constructed blocks used for the staining had ~100 tissues per block with duplicates across the two TMA blocks. A representative group of 92 tissue samples were included on both of the constructed TMA blocks.

### Staining by immunohistochemistry (IHC)

The TMA blocks were sectioned onto Flex IHC slides (Dako, Inc.), deparaffinized and hydrated, followed by antigen retrieval in the PT Linker (Dako, Inc.). The slides were stained for the indicated antibody and antigen retrieval conditions summarized in Additional file 1: Table S1. The IHC staining was processed on a Dako Cytomation Autostainer Plus. And followed with a Hematoxylin nuclear counterstain (Dako, Inc.). For quality control purposes, known positive control and negative control specimens were included for each antibody set.

### Image scanning and analysis

The slides were digitally scanned at a 200X magnification on an Aperio ScanScope CS whole slide scanner (Leica, Biosystems, Inc.). The generated digital images were saved onto the eSlide Manager database (ver. 12.0.1.5027).

To quantify the area of positive staining and density or the number of cells stained with DAB chromagen, customized macros for each stain were generated from the Color Deconvolution and Cell Quantification algorithms in the Aperio Image Analysis Tools software. All the cores and regions of interest on each TMA slide were labelled and submitted for analysis with a proper validated macro for each stain. The output results, included percentage of positively stained area and density or positive stained cell numbers of each intensity levels, respectively. The mark up core images were re-evaluated, and the generated data were exported from ImageScope annotation files as an Excel file for statistical analyses. The scored regions of each sample were checked manually to see if the algorithms had false positives or false negatives. The sample was not included if >10 % of the cells were misclassified.

### Statistical analysis

Cancer and non-cancer (“not cancer”) samples were compared by Mann-Whitney nonparametric analysis using Prism software. All the tests used a confidence interval of 95 % (α = 0.05).

To compare the ER status in our data and in METABRIC data [22] while taking age into account, we used the following logistic regression model:

$$ {\textstyle \mathrm{logit}}\left({\pi}_{{\textstyle \mathrm{E}\mathrm{R}\hbox{-} }}\right)={\beta}_0+{\beta}_1\cdot {\textstyle \mathrm{age}}+{\beta}_2\cdot I\left({\textstyle \mathrm{our}\ \mathrm{data}\ \mathrm{or}\ \mathrm{not}}\right) $$

In the model, ${\pi}_{{\textstyle \mathrm{E}\mathrm{R}\hbox{-} }}$ is the probability of ER-, age is the age of the patient at diagnosis, and *I*(our data or not) equals 1 if the patient is from our data, and 0 if the patient is from the METABRIC data. Note that this model means that for a patient in METABRIC data, logit(π_ER ‐_) = *β*_0_ + *β*_1_ ⋅ age, and for a patient in our data, logit(π_ER ‐_) = *β*_0_ + *β*_1_ ⋅ age + *β*_2_. Therefore, to test whether the ER status is different in the two datasets while taking age into account, we tested *H*_*0*_: *β*_2_ = 0 vs *H*_*A*_: *β*_2_ ≠ 0_._ The p-value was *P* = 0.11, which is not significant. This indicates that the different ER- proportions in the two datasets is likely caused by the age difference in the two populations. (Also, our model confirms that age has a very significant effect on ER status: *β*_1_ ≠ 0 with *p*-value < 1 × 10^−10^.)

## Results

### Young age at diagnosis for breast cancer patients in western Kenya

**Fig. 1:**
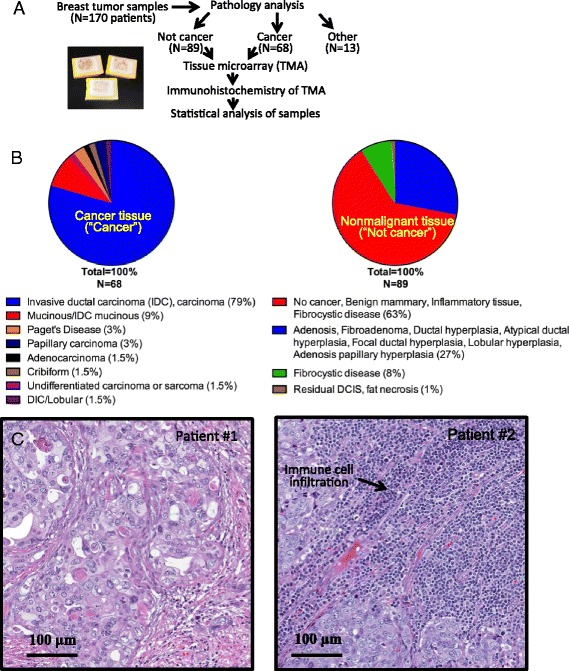
Pathology of Kenyan breast cancer tissue samples. **a** Experimental design flowchart for this study. Samples were collected, analyzed for pathology, processed to create a tissue microarray, stained for clinical marker immunohistochemistry, and quantified by statistical analysis. **b** Pie chart representations of the distribution of cancer (left) and benign/not cancer (right) pathologies in Kenyan breast tissues analyzed after H&E staining. Most of these patients were diagnosed with invasive ductal carcinoma (IDC) and mucinous IDC. Most benign samples fell into the category that includes benign mammary, inflammatory tissue, and fibrocystic disease. (C) H&E staining of representative Kenyan breast cancer samples analyzed for pathology. Both Patient 1 and Patient 2 have invasive ductal carcinoma (IDC). Patient 2 has significant immune cell infiltration

To characterize the breast cancer seen in western Kenya, we first collected, processed, and sectioned 170 primary breast tissue samples collected at the Moi Teaching and Referral Hospital, Eldoret, Kenya (Fig. 1a). For an initial pathological diagnosis based on morphological criteria, we used hematoxylin and eosin (H&E) stained tissue sections from each patient’s tumor tissue. From the pathology analysis, we grouped the patient tissues into cancer and not cancer categories. Based on this analysis, we excluded patient samples that were not breast tissue, were inconclusive, or were of low quality based on the pathology. The remaining samples included 68 cancer and 89 not cancer tumor tissue samples.

The Kenyan breast cancer patients who participated in this study included a diverse group of patients ranging from age 16 to 84 (Table 1). The median age at diagnosis for the Kenyan breast cancer patient cohort was 48.5 years, and the mean age was 51.9 years, which are both younger than the mean age at diagnosis of U.S.-born white breast cancer patients (64.1 years of age), U.S.-born black breast cancer patients (59.1 years), or Jamaica-born black breast cancer patients (56.5 years) who live in the U.S. [23]. This mean age at diagnosis of this Kenyan cohort is similar in age to both Western Africa-born black breast cancer patients (48 years) and Eastern-Africa born black breast cancer patients (48 years) who live in the U.S. [23].

Fifty-eight of the 68 patients with cancer provided gender information. The breast cancer patients were 93 % female and 7 % male (i.e., 4 of 58 cancer patients). The rate of male breast cancer is higher in this population than the one percent rate seen in the U.S. [24] and in other East African studies (Table 2) [17, 19, 25–29]. For additional analysis, we applied a Fisher’s exact test to test whether our study has a larger proportion of male breast cancer patient than is seen in other geographic regions. The percentage of male breast cancer patients was significantly different from other large breast cancer population studies from Tunisia, Nigeria, and the United States (N > 1437 patients) (Additional file 2: Table S2). This suggests that the difference in percentage of male patients is unlikely due to chance alone. We also compared our patient population with smaller studies collected in Kenya, Uganda, Tunisia, and Zimbabwe. While the number of male breast cancer patients seen in our Kenyan patient population did not reach statistical significance, the lack of statistical significance in these studies may be due to the smaller sample sizes.

**Table 2:** Comparison of breast cancer studies from East Africa

Study	Country	City	Patients with Breast Cancer	Retrospective or prospective study design	Female	Male	Ethnic groups considered in study	Immune cells quantified	Median	Mean	ER+	ER-	ER+	ER-	PR+	PR-	PR+	PR-	HER2+	HER2-	HER2+	HER2-	TN	TN	Time Period of Sample Collection	Date of Publication
			(N)^#^		(%)	(%)			age	age	(N)	(N)	(%)	(%)	(N)	(N)	(%)	(%)	(N)	(N)	(%)	(%)	(N)	(%)		
This Study, Sawe et al.	Kenya	Eldoret	48 (68)^A^	Prospective	93	7	Yes	CD68, CD163, CD4, CD8, CD20, CD25	48.5	51.9	29	20	59	41	19	29	40	60	7	42	14	86	16	33	2011–2013	
Nalwoga et al.	Uganda	Kampala	65^B^	Retrospective	100	no ^B^	No	None		49.8	23	42	35	65	15	50	23	77	19	46	29	71		34 ^G^	1993–2002	2007
Roy et al.	Uganda	Kampala	35 (45)^B,C^	Retrospective	96	4 ^C,B^	No	None			27	18	60	40					5	39	11	89	16	36	2000–2004	2011
Bird et al.	Kenya	Kijabe	34 (129)^D^	Prospective	97	3	No	None	47	48	29		24						9	25	26	74	15	44	2001–2007	2008
Nyagol et al.	Kenya	Nairobi	158^B^	Prospective	100	no ^B^	No	None		47	59	99	37	63	77	81	49	51	44	114	28	72	44	28	2002–2004	2006
Wata et al.	Kenya	Nairobi	54 (219)^B,E^	Retrospective	100	no ^B^	No	WBC, platelets	45	46.5	30	34	47	53	28	36	44	56	19	35	35	65			2007–2008	2013
Kantelhardt et al.	Ethiopia	Addis Ababa	352^B^	Retrospective	100	no ^B^	No	None		40.1-43^H^	230	122	65	35	176	168	51	49	n.d.	n.d.	n.d.	n.d.			2005–2010	2014
Burson et al.	Tanzania	Dar es Salaam	57 (488)^I^	Retrospective	97	3	No	None		49.4	33	32	51	49	29	36	45	55	n.d.	n.d.	n.d.	n.d.			2009–2010	2010

*ER* estrogen receptor, *PR* progesterone receptor, *HER2* human epidermal growth factor, *TN* triple negative (ER-,PR-,HER2-), *N* number, % = percent, *n.d* not determined

^#^The number before the parentheses is the number of patients used for analysis of receptor status and is summarized individually by the indicated superscripts

The number in parentheses represents the total number of patients with breast cancer in the study.

^A^ *N* = 48 patients with PR and triple negative data. *N* = 49 with ER, HER2 data. Excluded patients who did not provide consent or who had chemotherapy prior to surgery

^B^Only females included in study

^C^ *N* = 35 patients with triple negative data. *N* = 44 patients with HER2 data. 2 of 47 (4 %) patients were male but were excluded from the study

^D^ *N* = 34 patients with HER2/triple negative data. 120 patients with hormone receptor data

^E^ *N* = 54 patients with HER2 data. *N* = 64 patients with ER data. *N* = 64 for PR. Excluded if <18 yrs old, male, or if chart did not have a date of diagnosis

^F^No hormonal receptor data in this study

^G^Defined in this study by staining for Cytokeratin 5/6 and P-Cadherin as basal subtype markers, rather than ER, PR, and HER2

^H^Mean is 43 years old for ER+ and 40.1 years old for ER- patients

^I^ *N* = 57 patients with ER and PR data. No HER2 data for these patients

Moreover, the patients predominantly come from two ethnic groups (i.e., Luyha and Kelenjin), are married, and have no known familial history of breast cancer. More than half of the patients used either injected or pill contraceptives.

### Breast cancer pathologies are predominantly invasive ductal carcinoma

We next examined the pathologies of the breast tissue samples using H&E tissue sections from each tumor. Invasive ductal carcinoma was the predominant pathology seen in the malignant tumors (79 % of cancer tissues) (Fig. 1b). Additional pathologies represented in the patient population also included mucinous carcinoma, Paget’s disease, adenocarcinoma, invasive carcinoma, lobular carcinoma, invasive lobular carcinoma, papillary carcinoma, invasive cribiform carcinoma, and undifferentiated carcinoma or sarcoma. Some of these tumors had significant inflammatory infiltration or mucinous pathologies associated with the carcinoma (Fig. 1c).

The pathologies of the non-malignant tissues included normal breast tissue as well as fibroadenoma and adenosis, fibrocystic disease, ductal hyperplasia, atypical ductal hyperplasia, apocrine metaplasia (not cancer), intraductal papilloma, papillary hyperplasia, tubular adenoma, and lobular hyperplasia (Fig. 1b). Only one sample had the pathology of ductal carcinoma in situ.

### Kenyan breast cancer samples are HER2 negative and are heterogeneous for ER and PR expression

**Fig. 2:**
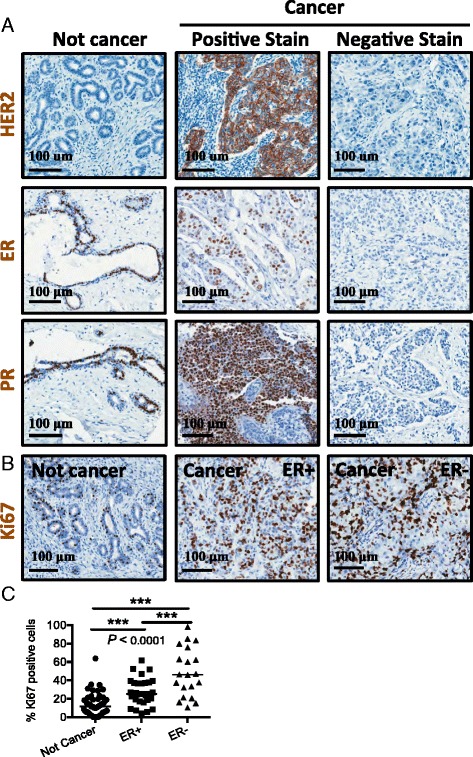
Heterogeneous expression of ER, PR, and HER2 receptors and increased proliferation. **a** Representative tissue samples from cancer and not cancer tissues that were stained for HER2, ER, and PR receptor expression. Examples of tissue that stained positively and negatively for the receptors are included. **b** Representative cancer and not cancer samples stained for the Ki67 proliferation marker. **c** Data plot analysis of Ki67 positive cells in ER+ vs. ER- tissue samples. Ki67 staining is significantly different between tissues from not cancer, ER+, and ER- breast samples (*P* < 0.0001, ANOVA) in ER- tissue samples, indicating high grade and an increase in cellular proliferation. The following combinations were significantly different by one-sided t-test: *P* = 2.834e-05 (ER+ vs. not cancer), 4.576e-06 (ER- vs. not cancer), and *P* = 0.0009 (ER- vs. ER+). The bar represents the median of all samples in the indicated cohort and includes any unstained samples

We next scored and quantified the clinical markers expressed in the breast tumor tissues collected for this study. We analyzed the patient tissue samples for expression of clinical markers of breast cancer (e.g., HER2, estrogen receptor/ER, and progesterone receptor/PR) using tissue microarrays (TMAs) we generated from the patient breast tissue blocks. We first stained and scored TMA sections for the receptor HER2 by immunohistochemistry (Fig. 2a). Eighty-six percent of the cancer samples were negative for HER2 expression. This distribution is similar to that seen in the USA and western countries.

We next stained TMA tissue sections by immunohistochemistry for estrogen receptor (ER) and progesterone receptor (PR) and scored the samples for positive expression of these receptors in the epithelium (Fig. 2a and Table 1). The majority of the cancers were ER positive (59 % ER positive vs. 41 % ER negative) and PR negative (60 % PR negative vs. 40 % PR positive). These rates are lower than those seen in western countries but could be a reflection of the cancers occurring in younger populations. To determine if the ER status was expected based on the age of the population, we statistically compared our dataset to another large breast cancer patient dataset (analysis described in Methods) (*N* = 1992 patients, METABRIC) [22]. Our analysis suggests that the differences in ER status of the two patient populations represented by the datasets likely are caused by the age difference in the two populations (i.e., the ER populations were not statistically different from each other; *P* = 0.11). In addition, our model also confirms that the age of the patient population has a very significant influence on the ER status (*P* < 1×10^10^).

### Cohort of patients with triple negative and highly proliferative breast cancer

We hypothesized that the western Kenyan cancers would also be enriched for triple negative breast cancer (HER2 negative, ER negative, PR negative). We compared the percentage of patients with triple negative breast cancer to the percentage of patients in other breast cancer studies. Indeed, we found a high representation (33 %) of triple negative breast cancer in the tissue samples (Table 1).

After determining the receptor status of the malignant samples, we next looked at proliferation in the non-cancer and cancer samples. Both cancer and non-cancer TMA tissue samples were stained for the proliferation marker Ki67 by immunohistochemistry and quantified for the percentage of Ki67 positive epithelial cells (Fig. 2b, c). The ER+ or ER- cancer tissues expressed more Ki67 positive cells than did the non-cancer samples. The following combinations were significantly higher in cancer samples compared to not cancer samples by one-sided *t*-test: *P* = 2.834e-05 (ER+ vs. not cancer) and 4.576e-06 (ER- vs. not cancer), respectively. In addition, both ER+ and ER- tumors expressed Ki67, with more proliferation in the ER- tumors than in the ER+ tumors (*P* = 0.0009 by one-sided *t*-test to test if ER- is larger than ER+; *P* = 0.002 by two-sided *t*-test to test if ER- is different from ER+). Because not only the ER- tumors but also the ER+ tumors expressed higher Ki67 than did not cancer tissue, this indicates that the tumors from the Kenyan patients are highly proliferative with a high grade.

### Increased infiltration of CD163+ M2 macrophages, CD25+ T regulatory cells, and CD4+ T helper cells, but not CD20+ B cells or CD8+ cytotoxic T cells, in Kenyan breast cancer tissue

**Fig. 3:**
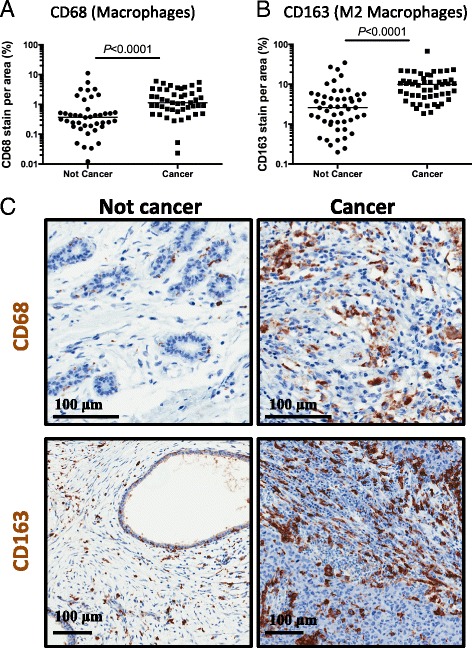
Kenyan breast cancer tissue samples have increased macrophage infiltration in primary breast tumors. **a** Data plot analysis of IHC analysis for the macrophage lineage utilizing a CD68 antibody. Quantitative analysis of the staining indicates a significant increase in percent of CD68+ stained area (*P* < 0.0001; Mann-Whitney). **b** Data plot of IHC analysis for the M2 macrophage lineage utilizing a CD163 antibody. Quantitative analysis of the IHC staining revealed a significant increase in percent of CD163 stained area (*P* ≤ 0.0001; Mann-Whitney) in M2 macrophages in cancerous Kenyan breast tissues versus noncancerous Kenyan breast tissues. **c** Immunohistochemistry of representative noncancer and cancer samples for both general macrophage lineage (CD68) and the M2 macrophage lineage (CD163). Because the graphs are a log scale, any samples with unstained sections (i.e., zero) are not included in the graph. The bar represents the median of all samples in the indicated cohort and includes any unstained samples

Since the analysis of the pathology of these tumors identified a large number of tumors with inflammatory cell infiltration, we wanted to identify which kinds of inflammatory cells were recruited to the tumor microenvironment during breast cancer progression. Macrophages, B cells, and T cells are among the most common leukocytes found in the stroma of neoplastic breast tissue [20, 30]. We stained the patient breast tissue samples for markers used to distinguish between these inflammatory cell types. We stained and scored patient tissue samples for CD68 (Fig. 3a, c), which is a macrophage marker, and CD163 (Fig. 3b, c), which stains M2 macrophages. The cancer tissue samples had increased CD68+ cells as well as increased M2 macrophage activation compared to the non-cancerous tissues. These results suggest that the cancer tissues have increased macrophage infiltration, marked by an increase in M2 macrophages.

**Fig. 4:**
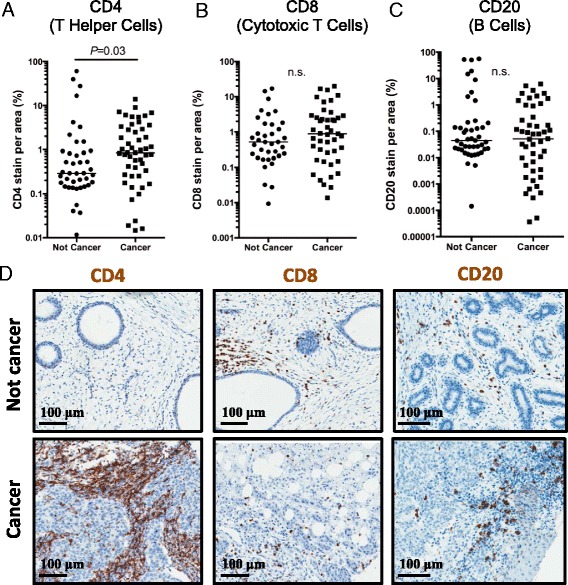
Distribution of CD4+, CD8+, and CD20+ cells in Kenyan breast cancer tissue samples. **a** Data analysis comparing the not cancer and cancer samples stained for T helper cell presence using a CD4 antibody. Significant increase was seen in T helper cell infiltration in the cancer samples shown by a higher percentage of CD4+ stained cell area (*P* = 0.03; Mann-Whitney). **b** Data analysis comparing noncancerous and cancerous samples stained for CD8+ cytotoxic T cells. No significant difference was seen in cytotoxic T cell infiltration in the cancerous samples, as shown by percentage of positively stained cell area (n.s.; Mann-Whitney). **c** Data analysis comparing the noncancerous to the cancerous samples stained for CD20+ B cells. No significant difference was seen in CD20+ cell infiltration in the cancerous samples, as shown by percentage of CD20+ stained cell area (n.s.; Mann-Whitney). **d** Immunohistochemistry of CD4, CD8, and CD20 in representative cancer and not cancer tissue samples.Because the graphs are log scale, any samples with unstained sections (i.e., zero) are not included in the graph. The bar represents the median of all samples in the indicated cohort and includes any unstained samples

To investigate the adaptive immune response in cancer, we stained and quantified the tissue samples for markers of both the cellular and humoral immune responses by immunohistochemistry. We stained tissues for CD4 (T helper cells), CD8 (cytotoxic T cells), and CD20 (B cell marker). Cancer tissues had increased recruitment of CD4+ T helper cells (Fig. 4a, d). In contrast, CD20 and CD8 positive cells were not differentially recruited to cancer versus non-cancer tissues (Fig. 4b–d).

Mature CD8+ T cells are cytotoxic effectors of the immune system that support antitumor activity and correlate with improved prognosis in breast cancer patients [31]. Because the CD8-mediated immune response was not sufficient to overcome the cancer cells in these patients, we hypothesized that the tumors developed an alternative strategy to overcome the cytotoxic activity of CD8+ T cells. Examples of immunosuppressive strategies used by tumor cells to escape immune surveillance include elevated activity of Treg cells to inhibit antitumor immune response, upregulation of CTLA-4 and PD-L1; TGF-ß production; and loss of T cell antigen presentation in the tumor (reviewed in [32]).

**Fig. 5:**
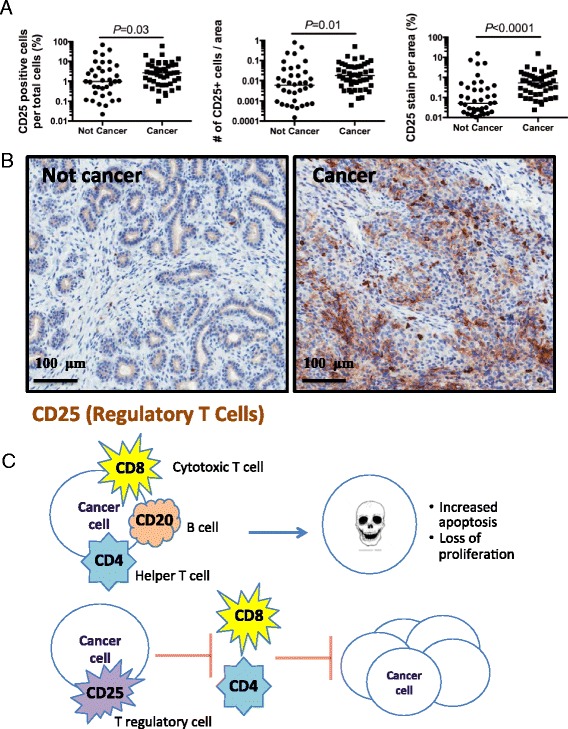
Increased infiltration of regulatory T cells in Kenyan breast cancer tissue. **a** Data analysis comparing the noncancerous and cancerous samples stained for CD25+ regulatory T cells. A significant increase was seen in regulatory T cell infiltration in the cancer samples, as shown by a higher percentage of positively stained cells (*P* = 0.03; Mann-Whitney), increased number of positively stained cells per area (*P* = 0.01; Mann-Whitney), and a higher percentage of CD25 stain per area (*P* = 0.0001; Mann-Whitney). Because the graph is a log scale, any samples with unstained sections (i.e., zero) are not included in the graph. The bar represents the median of all samples in the indicated cohort and includes any unstained samples. **b** Representative cancer and not cancer tissue samples stained for CD25. **c** Proposed T cell mechanism of action in Kenyan breast cancer model. (Top) Without a strong presence of regulatory T cells (e.g., benign Kenyan tissue), cytotoxic and T helper cells are able to combat and suppress the cancer cell, leading to increased apoptosis and loss of proliferation. (Bottom) When T regulatory cells are present (e.g., Kenyan breast cancer tissue), they block cytotoxic and helper T cells from fighting off the cancer cells

We examined the infiltration of CD25+ regulatory T cells (Tregs) into the tumor. A primary function of CD25+ Tregs is to inhibit the antitumor activity of CD8+ cytotoxic T cells [31]. Also, M2 macrophages, which are increased in these tumors (Fig. 3), can recruit Tregs [33]. We looked at the recruitment of Tregs by immunostaining the tissue samples for CD25. Interestingly, we saw an increase in the number of CD25+ cells recruited to the cancer compared to the non-cancer tissues (Fig. 5a, b). These data suggest that CD25+ Tregs are recruited to breast malignancies in a subset of the western Kenyan patient cohort and may impair their immune response to tumors in these patients.

## Discussion

In this prospective study, we characterized breast cancer tissue samples collected from consecutive patients of western Kenya. We found that a high percentage of these patients were diagnosed with cancer at a young age and developed a low three-year survival rate. The majority (59 %) of the breast tumors expressed estrogen receptor, while 33 % of the tumors were triple negative. The breast tumors were highly proliferative and high grade invasive ductal carcinomas with immune cell infiltration. The immune cells recruited to the tumor included cells expressing markers CD68, CD163, CD4, and CD25. Because a western Kenyan breast cancer patient population has not been studied previously, this study has important implications for identifying appropriate treatment strategies that are required to reduce mortality of Kenyan breast cancer patients, who currently have limited diagnostic and treatment opportunities.

### Estrogen receptor status

Though the majority of the breast tumors from our patient cohort were ER+, roughly 33 % of the breast cancer patients in our study had triple negative breast tumors, which also are indicative of poor prognosis. These results are consistent with other studies that find that 23 %-44 % of breast cancer tissue samples collected from East African women in Kenya, Ethiopia, and Uganda are triple negative [17, 19, 25–27, 34]. African women with breast cancer also have a higher prevalence of ER- and triple negative cancer compared with Caucasian populations [6, 14, 19, 34, 35]. These results also are similar to that seen in breast tumors from black women in the U.S. and in the United Kingdom, as compared to white women, where the patient cohorts also had a high representation of triple negative/basal subtype breast tumors [36, 37].

### Treatment of breast cancer in Western Kenya

Testing for ER status is not a standard test for breast cancer treatment in Kenya but would provide a significant advancement in directing the treatment strategy of these patients. When markers have been used to direct chemotherapy and hormone therapies as treatment strategies, the patients have had improved survival and reduced metastasis rates [28]. Unfortunately, because of limited resources in Kenya, clinical marker testing and treatments for these patients are particularly challenging [1, 6].

Since the majority of the tumors in this study were ER+, this suggests that the majority of these western Kenyan patients are candidates for treatment with hormone therapy, such as tamoxifen, fulvestrant, or aromatase inhibitors. In contrast, since almost all ER-tumors were triple negative, the patients with ER-tumors instead should be treated with chemotherapy and/or radiation. However, the lack of facilities for chemotherapy and radiation make it imperative that efforts should be focused on early detection by community education and screening. For example, Moi Teaching and Referral Hospital in Eldoret, Kenya, where this study was initiated, has standard of care for breast cancer patients that predominantly includes surgical procedures/mastectomy and chemotherapy (personal observation). Because this hospital currently does not have a radiotherapy machine, radiotherapy is not even an option for these patients without additional resources and significant travel [38].

### Ethnic groups and regional differences in Western Kenya

Our study uniquely includes ethnic group information on its patient population. The breast cancer patients in this study have a different genetic background from population in other regions throughout Kenya based on the ethnic population data. The Uasin Gishu County website suggests that this county is “largely a cosmopolitan region, with the Nandi people of indigenous Kalenjin communities having the highest settlement.” Similar to the county data, the patients from MTRH in our study are predominantly in two ethnic groups: Luyha (38 %) and Kalenjin (34 %). In contrast, nationally the Kikuyu ethnic group is ranked first (22 %), followed by Luhya (14 %), and Kalenjin (12 %). No ethnic group data were included in the other breast cancer studies including patients from East Africa. However, the Nairobi ethnic group demographics differ from Eldoret ethnic group demographics and likely are reflected in their patient populations.

### Male breast cancer

We uniquely included men who developed breast cancer in the analysis of our study and found a rate of 7 % male breast cancer. This rate of male breast cancer is very high and is unusual. While most of the studies on East Africa patients excluded male patients from their analysis [17, 25–28], three other East Africa studies reported a high rate of male breast cancer (3–4 %) but have a lower rate compared to our study [19, 26, 29]. The percentage of male breast cancer patients was significantly different from other large breast cancer population studies from Tunisia, Nigeria, and the United States (Additional file 2: Table S2). This suggests that the high percentage of male patients in Eldoret patients is unlikely due to chance alone.

### Immune cell infiltration

To our knowledge, before this study, immune cell distribution by immunohistochemistry has not been studied in any other patient cohorts from Africa, making this study significant. Infiltration of macrophages, B cells, and T cells often increases with and are required for pathological breast cancer progression (reviewed in [20, 31, 39, 40]). Infiltration of mouse breast tumors with macrophages leads to an increase in breast cancer progression and lung metastasis, while depleting macrophages reduces them [40]. In addition, transgenic mouse models of breast cancer that are deficient in CD4+ cells initially developed primary tumors at similar rates but developed lung metastasis at a lower frequency than wildtype mice, suggesting that CD4 + T cells are required for breast cancer during lung metastasis [20]. These leukocytes also contribute to therapeutic response. Interestingly, cytotoxic drugs themselves can trigger tumor cells to release macrophage/monocyte recruitment factors, which in turn promote the infiltration of tumor-associated macrophages to the tumor cells. In addition, when macrophage recruitment is blocked with antagonists to colony stimulating factor 1 receptor (CSF1R) and in combination with paclitaxel, this treatment causes both an increase in primary breast tumor and lung metastasis as well as increased tumor suppression that was CD8+ CTL cytotoxic T cell-dependent [39].

In our study, the breast cancer tissue samples tested had increased T cell and macrophage immune cell marker (CD4, CD25, CD63, CD163) expression compared to benign tissue. M2 macrophages (CD163+) and regulatory T cells (CD25+) in particular are associated with pro-tumor roles during breast cancer progression. In fact, M2 macrophages and Tregs have a complimentary and synergistic relationship to promote their plasticity [41]. Tregs can differentiate monocytes/macrophages into CD163+ M2 macrophages [42]. M2 macrophages also can secrete chemokines that promote the induction, differentiation, and recruitment of Tregs [33].

In normal tissues, Tregs prevent autoimmune responses; in tumors, Tregs prevent the destruction of tumors by CD8+ cytotoxic T cells and contribute to cancer cells evading their detection by the immune system (Fig. 5c) [31]. Moreover, infiltration of Tregs into breast tumors is prognostic of reduced survival in patients [21]. Conversely, therapeutic strategies that eliminate the Tregs to modulate Treg activity (e.g., anti-CD25 mAb and CTLA-4 antagonists) have had some success in treating melanoma patients [43]. The Kenyan cancer patients with tumors that express high CD25+ cells might benefit from a similar therapeutic strategy designed to overcome the inhibitory immune response and to improve the anti-tumor immune response for patients with high levels of Tregs within the tumor site. Future research will be required to determine if immune modulators that eliminate Tregs or increase tumor destruction by CD8+ T cells will help in overcoming the aggressive breast cancer seen in Kenya.

### Patient population of this study

With our current sample size, our study identified both clinical features (e.g., male breast cancer, young age at presentation, estrogen receptor status) and immune cell features of this patient population that are worthy of additional studies. Our breast cancer patient sample size that was used for hormone receptor status (*N* = 48 breast cancer tissue with known hormone receptor status of breast cancer of 68 total patients) is comparable to other breast cancer studies from Kenya, Uganda, and Tanzania: (*N* = 34 with hormone receptor status of 129 total patients; [19]); (*N* = 54 with hormone receptor status of 219 total patients; [28]); (*N* = 158; [27]); (*N* = 35 patients with hormone receptor status of 45 total patients enrolled in study; [26]); (*N* = 65; [25]); and (*N* = 57 with hormone receptor status of 488 total patients; [29]). In fact, a large meta-analysis of African breast cancer studies that included receptor status did not find a small study bias that affected the receptor status in the studies analyzed from sub-Saharan Africa, which includes Kenya [44].

Of the studies mentioned above, only two were prospective studies [19, 27]. Older retrospective samples trended to have a lower percentage of ER+ tumors compared to prospective studies [44]. In a meta-analysis of studies from Sub-Sahara Africa, tumor tissue samples from retrospective studies included 10 % less ER+ tumors compared to prospective studies, suggesting increased bias in retrospective studies [44]. Our study was strengthened by being a prospective study and by not being a convenience sample.

### Changing landscape of breast cancer in Africa

Between 1980–2010, breast cancer incidence and death increased in Kenya and throughout Africa [2]. The cause of this increase in breast cancer is unknown but is speculated to be associated with risk factors, such as obesity, the increased awareness and detection of breast cancer, and the aging population [2, 3, 6]. However, an environmental factor that contributes to the aggressive nature of the disease progression would also be consistent with the results of our study, which include the young age at the presentation of the disease, the high percentage of males with breast cancer, and the suppressed antitumor immune response.

Interestingly, in contrast to our study, the prevalence of ER-negative breast cancer in East African-born blacks who immigrate to the U.S. is similar to the prevalence seen in U.S.-born whites [23]. However, the study’s East African-born population primarily was born in Ethiopia or Eritrea and may not represent the western Kenya patient population in our study. In contrast to this East African-born population, the ER-negative breast cancer in West African-born blacks is similar to the prevalence in U.S.-born black women. This finding suggests differences in the disease progression between black populations born in West or East Africa. Because these differences are not apparent when populations remain in Africa, the differences in prevalence are likely to be caused by a combination of environmental and genetic factors. Because of the small number of patients who donated detailed clinical data, our study is inconclusive to determine if these factors impact or affect the risk of breast cancer in Kenyan breast cancer. In future studies, we will track these patients over time to define more clearly which patients have the worst prognosis. Such studies will be important to identify the mortality rate of the patients, since many countries, including those in sub-Saharan Africa, do not have complete mortality data and instead must estimate mortality rates [2].

## Conclusions

Our data suggest that the breast cancer developing in patients of western Kenya is aggressive and is associated with diagnosis at a young age, high proliferative index and high immune cell infiltration in the primary tumors, and poor three-year patient survival. Our characterization of this patient population using clinical markers suggests possible treatment strategies that will be effective in improving the outcome for these patients. This research enhances our ability to diagnose and treat breast cancer patients in Kenya. In addition, achievements in our understanding of the etiology and treatment of a unique population of Kenyan patients with aggressive breast cancer may help identify and treat other poor prognostic breast cancers from other populations with a similar cancer disease progression.

## Additional files

Additional file 1: Table S1. Antibodies used in this study. (PDF 42 kb) Additional file 2: Table S2. Comparison of gender rates in breast cancer patients of Eldoret, Kenya and other regions. (PDF 73 kb)

## Abbreviations

ER: estrogen receptor
Her2/neu receptor HER2: human epidermal growth factor 2 receptor
IHC: immunohistochemistry
MTRH: Moi Teaching and Referral Hospital
PR: progesterone receptor
TMAs: tissue microarrays
Tregs: regulatory T cells
