# Supplementary material for: Aggressive breast cancer in western Kenya has early onset, high proliferation, and immune cell infiltration
Source: BMC Cancer. 2016 Mar 10;16:204. doi: 10.1186/s12885-016-2204-6 (PMC4787041; doi:10.1186/s12885-016-2204-6)
Supplement: Additional file 1: Table S1. — Antibodies used in this study. (PDF 42 kb) [file 12885_2016_2204_MOESM1_ESM.pdf]

Table S1. Antibodies used in this study

Antibody name	Antigen	Ab source	Positive control	Company	Catalog #	Dilution*	Special Methods
HER2	Her2 protein	rabbit	breast	sk001	90456	RTU	
ER alpha	Estrogen receptor	rabbit	breast	IRO84 DAKO	10075784	RTU	
PR	Progesterone receptor	mouse	breast	IR068 DAKO	10075666	RTU	
Ki67	Expressed during all active phases of cell cycle but absent in resting cells	mouse	tonsil	IR626 DAKO	93901	RTU	
CD68	Macrophages	mouse	tonsil	IR609 DAKO	85199	RTU	
CD163	M2 macrophages	mouse	bone marrow	VP-C374	6017007	1/100	
CD4	Thymocytes, T helper cells, and peripheral T cells	mouse	tonsil	IR649 DAKO	86211	RTU	no H202
CD8	Cytotoxic/suppressor T cells	mouse	tonsil	IR623 DAKO	87824	RTU	
CD20	B cells	mouse	tonsil	IR604 DAKO	92175	RTU	
CD25	Regulatory T cells. Type 1 transmembrane protein on activated B, T cells and some thymocytes.	mouse	tonsil	SK200 DAKO	10067590	RTU	

*RTU=ready to use
