# Supplementary material for: Aggressive breast cancer in western Kenya has early onset, high proliferation, and immune cell infiltration
Source: BMC Cancer. 2016 Mar 10;16:204. doi: 10.1186/s12885-016-2204-6 (PMC4787041; doi:10.1186/s12885-016-2204-6)
Supplement: Additional file 2: Table S2. — Comparison of gender rates in breast cancer patients of Eldoret, Kenya and other regions. (PDF 73 kb) [file 12885_2016_2204_MOESM2_ESM.pdf]

Table S2. Comparison of gender rates in breast cancer patients of Eldoret, Kenya and other regions.

Country	Males (N)	Males (%)	Females (N)	Females (%)	Total (N)	P value, compared to current study	References
Kenya	4	7	54	93	58		This study
Kenya	4	3	125	97	129	0.2086	Bird et al.[a]
Uganda	2	4	45	96	47	0.444	Roy et al.[b]
Nigeria	7	9	72	91	79	0.7662	Ogundiran et al.
Zimbabwe	2	2	124	98	126	0.0798	Chokunonga et al.
Tunisia	29	2	1408	98	1437	**0.036**	Maalej et al.
Nigeria	26	2	1287	98	1313	**0.0347**	Ezeome et al.
United States	2350	1	231,840	99	234190	**0.0028***	Siegel et al.

*In contrast to our current study, an analysis comparing two East African populations (Bird *et* al.; Roy *et al*.) to Siegel *et al.* suggests that neither patient population differs as significantly from Siegel (P-values compared to Siegel are 0.081[a] and 0.041[b], respectively).
